# Effects of Alpha-Connexin Carboxyl-Terminal Peptide (aCT1) and Bowman-Birk Protease Inhibitor (BBI) on Canine Oral Mucosal Melanoma (OMM) Cells

**DOI:** 10.3389/fvets.2021.670451

**Published:** 2021-06-10

**Authors:** Ayami Sato, Ivone Izabel Mackowiak da Fonseca, Márcia Kazumi Nagamine, Gabriela Fernandes de Toledo, Rennan Olio, Francisco Javier Hernandez-Blazquez, Tomohiro Yano, Elizabeth Shinmay Yeh, Maria Lucia Zaidan Dagli

**Affiliations:** ^1^School of Veterinary Medicine and Animal Science of the University of São Paulo, São Paulo, Brazil; ^2^Institute of Life Innovation Studies, Toyo University, Tokyo, Japan; ^3^Department of Pharmacology and Toxicology, Simon Comprehensive Cancer Center, School of Medicine, Indiana University, Indianapolis, IN, United States

**Keywords:** melanoma, connexin, peptide, viability, aCT1, Bowman-Birk inhibitor

## Abstract

Oral mucosal melanomas (OMM) are aggressive cancers in dogs, and are good models for human OMM. Gap junctions are composed of connexin units, which may have altered expression patterns and/or subcellular localization in cancer cells. Cell-to-cell communication by gap junctions is often impaired in cancer cells, including in melanomas. Meanwhile, the upregulated expression of the gap junction protein connexin 43 (Cx43) inhibits melanoma progression. The α-connexin carboxyl-terminal (aCT1) peptide reportedly maintains Cx43 expression and cell-cell communication in human mammary cells and increases the communication activity through gap junctions in functional assays, therefore causing decreased cell proliferation. The Bowman-Birk protease inhibitor (BBI), a component of soybeans, induces Cx43 expression in several tumor cells as a trypsin–chymotrypsin inhibition function, with antineoplastic effects. This study investigated the effect of aCT1 peptide and BBI treatment, alone or in combination, on TLM1 canine melanoma cell viability. Cell viability after treatment with aCT1, the reverse sequence peptide (R-pep), and/or BBI for 5 days was analyzed by PrestoBlue assay. Immunofluorescence was used to observe Cx43 localization and expression. aCT1 (200 μM) alone did not significantly decrease cell viability in TLM1 cells, whereas BBI (400 μg/ml) alone significantly decreased the TLM1 viability. Combined treatment with both aCT1 (200 μM) and BBI (400 μg/ml) significantly decreased cell viability in TLM1 cells. Cx43 expression, as identified by immunostainings in TLM1 cells, was increased in the cell membrane after the combination treatment with BBI and aCT1. This dual treatment can be combined to achieve the anticancer activity, possibly by increasing Cx 43 expression and affecting Cx43 migration to the cell membrane. In conclusion, a treatment strategy targeting Cx43 with BBI and aCT1 may possibly lead to new effective therapies for canine OMM.

## Introduction

Melanoma is an aggressive skin and mucosal cancer that develops from melanocytes. This tumor arises due to random genetic mutations, and after the melanoma has spread, it rapidly becomes life-threatening (1). In humans, the diagnosis of early-stage melanomas can facilitate their cure by surgical resection, and approximately 80% of cases are treated in this manner. However, metastatic melanoma is largely refractory to the existing therapies and has a very poor prognosis, being the survival rate for 5-years lower than 15% of the cases (2). Therefore, new treatment strategies are urgently needed. Continued research into more effective therapies for melanoma will improve the treatment and prognosis of these patients.

Canine oral and mucosal melanoma (OMM) are considered good models of human OMM, because they share many similarities including morphology, genetic alterations, and behavior (3–6). OMM is one of the most common oral malignancies in canines. OMM in dogs are considered extremely aggressive tumors, with local invasiveness and high metastatic propensity. The World Health Organization staging scheme for dogs with OMM is based on the size of the tumors (7). MacEwen et al. (8) correlated these stages with survival times. Stage I tumors, with <2 cm diameter has a median survival after surgery of 17 to 18 months. Stage II OMM are 2 to <4 cm in diameter tumors, and the survival time is 5 to 6 months. In stage III tumors of ≥4 cm in diameter and/or lymph node metastasis, the median survival is 3 months. Stage IV dogs with OMM have distant metastasis and the prognosis is very poor (9). Therefore, some factors negatively affect the prognosis including the clinical stage, tumor size, evidence of metastasis, and reported histologic criteria for melanoma prognosis. Standardized treatments, such as surgery, radiotherapy, and chemotherapy, have provided minimal to modest stage-dependent clinical benefits, and death in general occurs due to metastasis (9). Notably, most of the medicines used in veterinary medicine are repurposed from drugs indicated for human use and are not being developed specifically for animals. Therefore, it is necessary to intensify the research focus on veterinary oncology, simultaneously testing possible therapeutic alternatives in animal studies and human trials.

Teixeira et al. previously found that canine amelanotic OMM present higher aggressive behavior than their melanotic OMM counterparts (10). This finding could be partly explained by the decreased expression of connexin 43 (Cx43), which probably resulted in an impaired cell-to-cell communication capacity and, consequently, greater cell proliferation. Regarding cell to cell communication, the expression of connexins could be an essential target factor in canine oral melanoma because Cx26 and Cx43 were significantly reduced in amelanotic melanomas (10).

Connexins are integral membrane proteins that form gap junctions or channels between adjacent cells, thereby permitting the bidirectional cytosolic exchange of ions, metabolites, and secondary messengers (<1,200 Da). These channels assemble into distinct plasma membrane structures termed gap junctions, and the intercellular communication at the gap junctions play important roles in tissue homeostasis and the regulation of cell growth and differentiation. Additionally, connexins form functional channels (i.e., hemichannels) in the non-junctional areas of the plasma membrane. These hemichannels provide a communication pathway between the intracellular and extracellular milieu, critical for autocrine and paracrine signaling. In addition, connexins present significant channel-independent roles, including their function as signaling hubs; these may occur at the plasma membrane, in the cytoplasm, or even in the nucleus (11). The connexin protein family in humans has 21 members, in which Cx43, named because of its molecular weight of 43 kDa, is the most extensively studied. Very few studies are available on connexins in canine tissues. Cruciani and Mikalsen (12), found the 18 “multi-specie” connexin genes (connexins 26,29/31.3, 30, 30.2/31.9, 30.3, 31, 31.1, 32, 36, 37, 39/40.1, 40, 43, 45, 44/46, 47, 50, and 57/62) in dogs. The expression of connexins in canine cancers has been evaluated in mammary tumors (13–15), bone tumors (16, 17) testes (18), and OMM (10).

Decreased or diminished expression and/or function of connexins have been observed in most tumor cell lines and solid tissue tumors, including melanomas (11). The role of gap junctions in tumor progression has been studied mainly through the ectopic reintroduction of connexin genes into tumor cell lines. The expression patterns of Cx43 have been studied in several cancer types in humans, and it varies depending on the cancer type and stage (19). The ectopic expression of Cx43 has been shown to reduce cell proliferation in many distinct cancer cells, including in mouse melanoma cell lines (20).

The overexpression of Cx43 reduces the proliferative and metastatic capacities of melanoma in mice (20), while the suppression of Cx43 expression by miR-106a promotes melanoma cell proliferation (21) in human-derived cells in *in vitro* studies. Furthermore, Cx43 upregulation is potentially able to inhibit melanoma progression in mice, as shown in an *in vivo* study (22). Therefore, the regulation of Cx43 expression may lead to the developing of an effective treatment strategy for melanomas; scientific evidence suggests that connexins could be an important therapeutic target (11, 19, 23–30).

The alpha-connexin carboxyl-terminal (aCT1) peptide is a 25-amino acid peptide that mimics the carboxyl-terminal of Cx43. At the molecular level, the aCT1 peptide inhibits the activity of Cx43 hemichannels by inducing their sequestration from the perinexus region surrounding the gap junctions, thereby reducing hemichannel density and availability for activation within the cell membrane (31). The aCT1 peptide is expected to clinically improve postsurgical scarring (32). Grek et al. suggested that using aCT1 peptide for targeting the gap junctional distribution and activity of Cx43 is an effective therapeutic strategy in human breast cancer. Furthermore, they demonstrated that aCT1 peptide enhances the activity of therapies like tamoxifen and lapatinib, thereby supporting the clinical potential of combinational strategies, including the modulation of Cx43 by the aCT1 peptide (33). In addition, Murphy et al. (34) indicated that combining aCT1 with temozolomide, an antineoplastic agent, could enhance the therapeutic responses in human glioblastoma cell lines. These findings suggest the possibility of a Cx43 targeting therapy, using aCT1.

Protease inhibitors are known cancer chemopreventive agents, because of their well-established *in vivo* and *in vitro* anti-carcinogenic activity in cancer models (35). The Bowman-Birk inhibitor (BBI) is the most predominant protease inhibitor in soybeans. It consists of a 71-amino acid protein (8 kDa), and a serine protease inhibitor, which has both trypsin and chymotrypsin inhibitory activities (36). BBI is a small water-soluble protein that is present in soybean and almost all monocotyledonous and dicotyledonous seeds, and BBI decreases the proteolytic activities of trypsin, chymotrypsin, elastase, cathepsin G, chymase, serine protease-dependent matrix metalloproteinases, urokinase protein activator, mitogen-activated protein kinase, and phosphoinositide 3 kinase (PI3K), and upregulates Cx43 expression. BBI was found to be an efficient suppressor of carcinogenesis (37). BBI has cancer-protective activities, although its exact mechanism(s) of action is incompletely understood. In previous studies, it was shown that Cx43 induction by BBI contributes to the decreased growth of tumor cells, both *in vivo* and *in vitro* (38, 39).

Therefore, we aimed to investigate the effect of aCT1 peptide and BBI on canine OMM cell viability and evaluate the usefulness of a Cx43-targeting strategy for the treatment of this cancer in dogs.

## Materials and Methods

### Ethical Statement

The study was submitted and approved by the Committee on Ethics on the Use of Animals (CEUA) of the School of Veterinary Medicine and Animal Science of the University of São Paulo, under protocol number 6968020817.

### Reagents

The aCT1 peptide and its reverse sequence peptide (R-pep) were synthesized by the American Peptide Company (Sunnyvale, CA). The aCT1 peptide is a short sequence at the Cx43 C-terminus, and this is linked to an antennapedia internalization sequence (RQPKIWFPNRRKPWKKRPRPDDLEI). The antennapedia internalization peptide sequence is RQPKIWFPNRRKPWKK. The R-pep sequence consists of the reverse sequence of aCT1 attached to an antennapedia sequence for internalization (33).

BBI was obtained from Sigma-Aldrich (#T9777, St. Louis, MO). All culture reagents were purchased from Thermo Fisher Scientific (Waltham, MA), unless otherwise indicated. The primary antibody, Purified Mouse Anti-Connexin 43, was purchased from BD Transduction Laboratories (#610061, San Jose, CA). The secondary antibody, Goat anti-mouse IgG (H+L) linked to Alexa fluor 488, was purchased from Invitrogen (#A28175, Waltham, MA).

### Cell Lines

The TLM1 canine oral melanoma cell line was kindly supplied by Dr. Jaime F. Modiano, VMD, PhD (University of Minnesota, Minneapolis, USA). The TLM1 cells originated from a canine (Gordon setter) oral melanoma. The cell lines were grown in Dulbecco's Modified Eagle Medium (DMEM, #12800-058) supplemented with 10% fetal bovine serum (FBS) and 1% Antibiotic–Antimycotic at 37°C in a humidified atmosphere with 5% CO_2_.

### Cell Viability Assay

**Figure 1:**
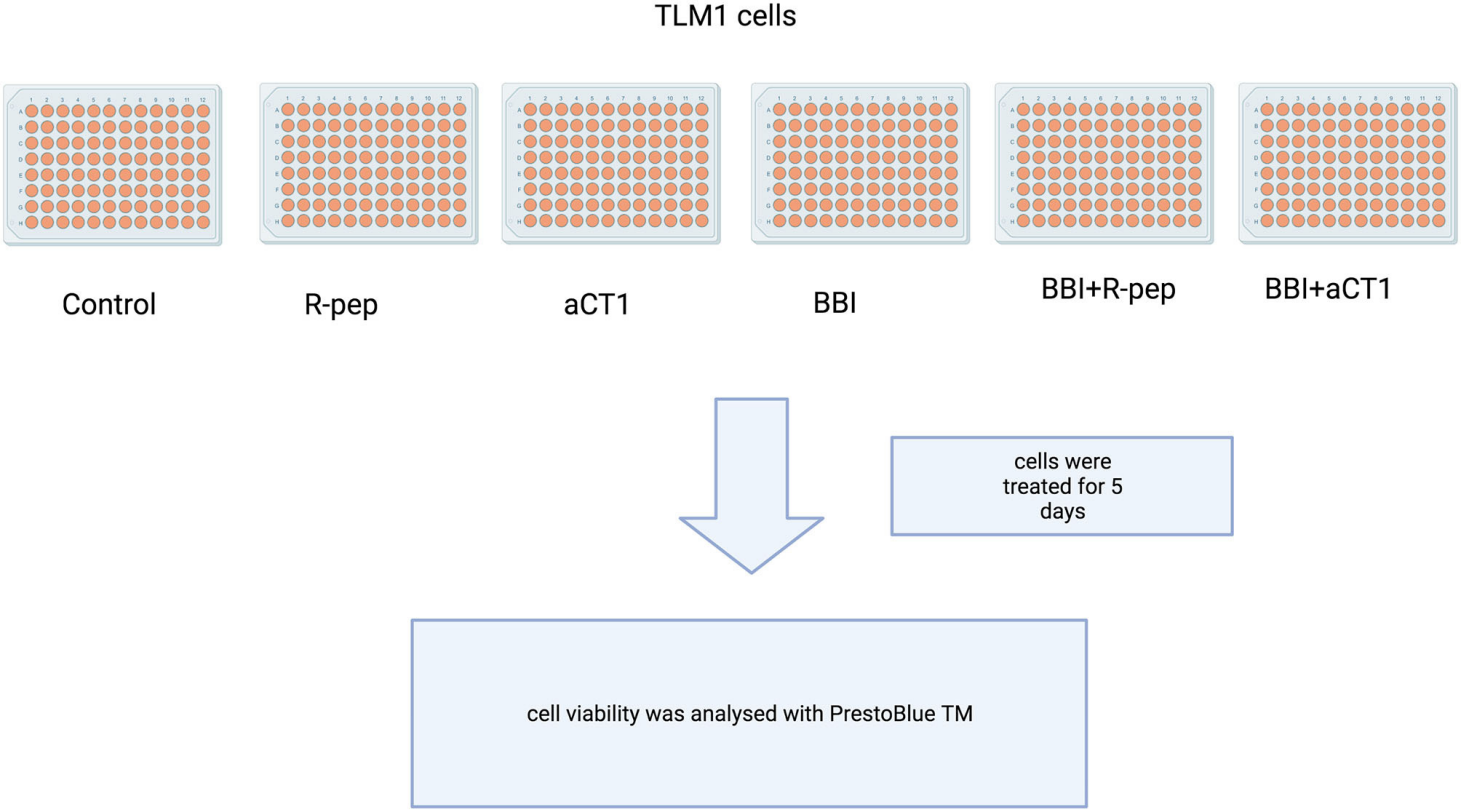
Scheme of TLM1 cells treatment with aCT1, BBI, BBI+R-pep, and BBI+aCT1, R-prep or control.

Cells (2 × 10^3^ cells/well) were seeded in culture plates with 96 wells with DMEM, containing 1% FBS, and cultured for 1 day. After incubation with each of the treatment reagents (i.e., Control, R-pep, aCT1, BBI, BBI+R-pep, and BBI+aCT1) for 5 days, cell viability was determined using PrestoBlue^TM^ Cell Viability reagent (#A13261, Thermo Fisher Scientific). Non-toxic resazurin in the PrestoBlue was converted to red-fluorescent dye within viable cells. The fluorescence at 570 nm was measured using a microplate reader (Figure 1).

### Immunofluorescence

In a 24-well plate, round coverslips of 12-mm diameter were placed in each well and prepared by irradiation using ultraviolet light for 15 min. Each cell line (1 × 10^5^ cells/well) was seeded in the plate with DMEM, containing 1% FBS, and cultured at 37°C in a humidified atmosphere with 5% CO_2_ for a day. Following the removal of the culture medium, 0.5 ml DMEM, containing aCT1 peptide or R-pep and/or BBI was added, and the plate was incubated at 37°C for 3 days. After washing with phosphate-buffered saline (PBS) two times, the cells were fixed by 0.5 ml 4% paraformaldehyde solution for 40 min at 4°C. The fixed cell samples were permeabilized with PBS with Tween 20 (PBS-T), 0.1% Triton X-100 and 5% Skim milk dissolved in PBS, for 30 min at room temperature (RT). Then, 100 μl/well of primary antibody solution (1:100 dissolved in PBS-T) was added to the cell sample. After leaving at 4°C overnight, PBS wash was undertaken three times. 100 μl/well of the secondary antibody solution (1:100 dissolved in PBS-T) was added to the cell sample. After leaving it at RT for 1.5 h in the dark, PBS wash was done three times. VECTASHIELD Antifade Mounting Medium with DAPI (#H1200, Vector Laboratories, Burlingame, CA) was dropped on each glass slide, and coverslips were placed on it. The slides were left overnight at 4°C in the dark to dye the cells with DAPI. Fluorescence microscopy, using ECLIPSE E800 (Nikon, Japan) with a setting of 40 × lens, was undertaken, and stained cytoplasmic membranes and nuclei were imaged (.jpeg), and the resulting images were merged using Image J software (Bethesda, MD).

### Statistical Analysis

Differences in group means were analyzed by one-way analysis of variance (ANOVA) followed by Tukey's test or Dunnett's. The GraphPad Prism 6 (GraphPad Software Inc., San Diego, CA, USA) was used for these calculations. *P*-values < 0.05 were considered significant.

## Results

**Figure 2:**
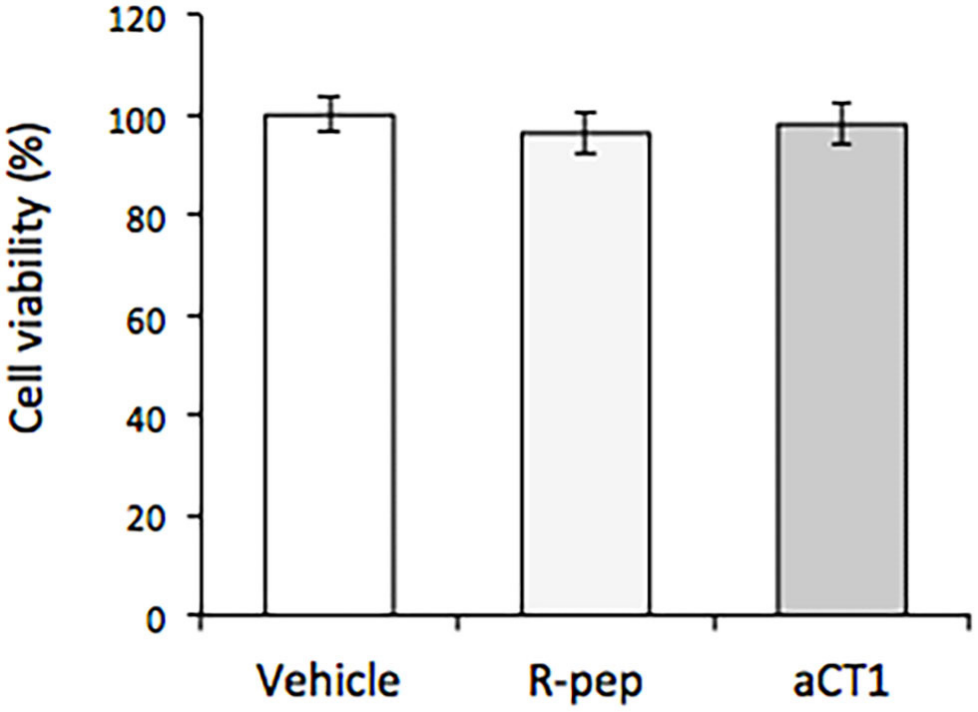
Effect of aCT1 peptide on cell viability in TLM1 cells. The cells were treated with 200 μM aCT1 or the reverse sequence peptide (R-pep) for 5 days. Cell viability was evaluated by a PrestoBlue assay. Columns represent means ± standard deviations (*n* = 5).

The effects of aCT1 and R-pep on cell viability in TLM1 cells were evaluated. Vehicle (water) was used to treat the control group. As shown in Figure 2, aCT1 peptide alone, or the R-prep, showed no effect on cell viability in TLM1 cells.

**Figure 3:**
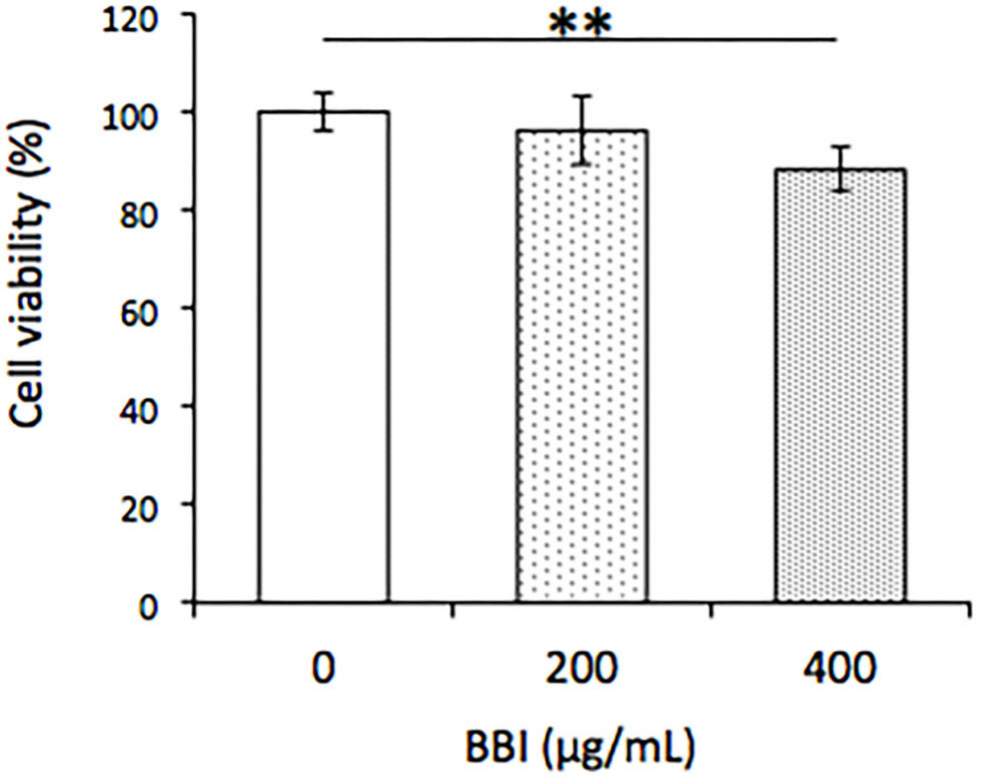
Effect of BBI on cell viability in TLM1 cells. The cells were treated with indicated concentrations of BBI for 5 days. Cell viability was evaluated by a PrestoBlue assay. Columns represent means ± standard deviations (*n* = 6). **p < 0.01 vs. 0 μg/ml, using Dunnett's test.

The effects of different concentrations of BBI (0, 200, and 400 μg/ml) on cell viability in TLM1 cells were evaluated. As shown in Figure 3, 400 μg/ml BBI treatment significantly decreased cell viability in TLM1 cells.

**Figure 4:**
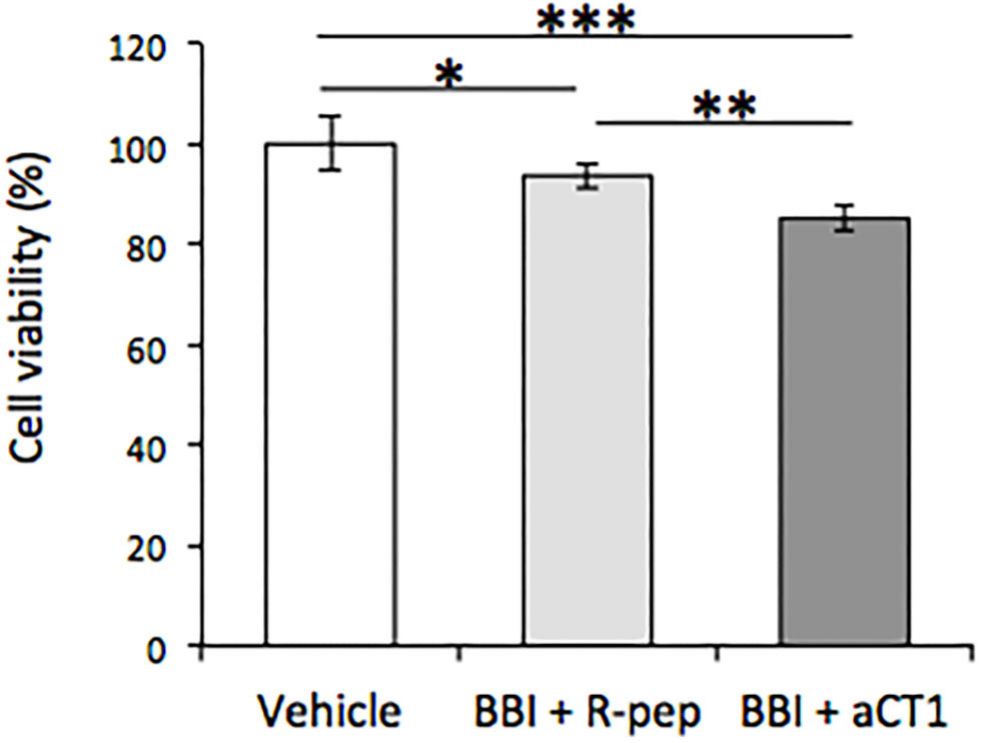
Effect of combination treatment of BBI and aCT1 peptide on cell viability in TLM1 cells. The cells were treated with 400 μg/ml BBI and 200 μM aCT1 (or R-pep) for 5 days. Cell viability was evaluated by a PrestoBlue assay. Columns represent means ± standard deviations (*n* = 6). *p < 0.05, **p < 0.01, ***p < 0.001, using Tukey's test.

Subsequently, the effects of combination treatment with BBI and aCT1 or R-pep were evaluated. As shown in Figure 4, combination treatment with BBI and aCT1 significantly decreased cell viability compared with combination BBI and R-pep treatment in the TLM1 cells.

**Figure 5:**
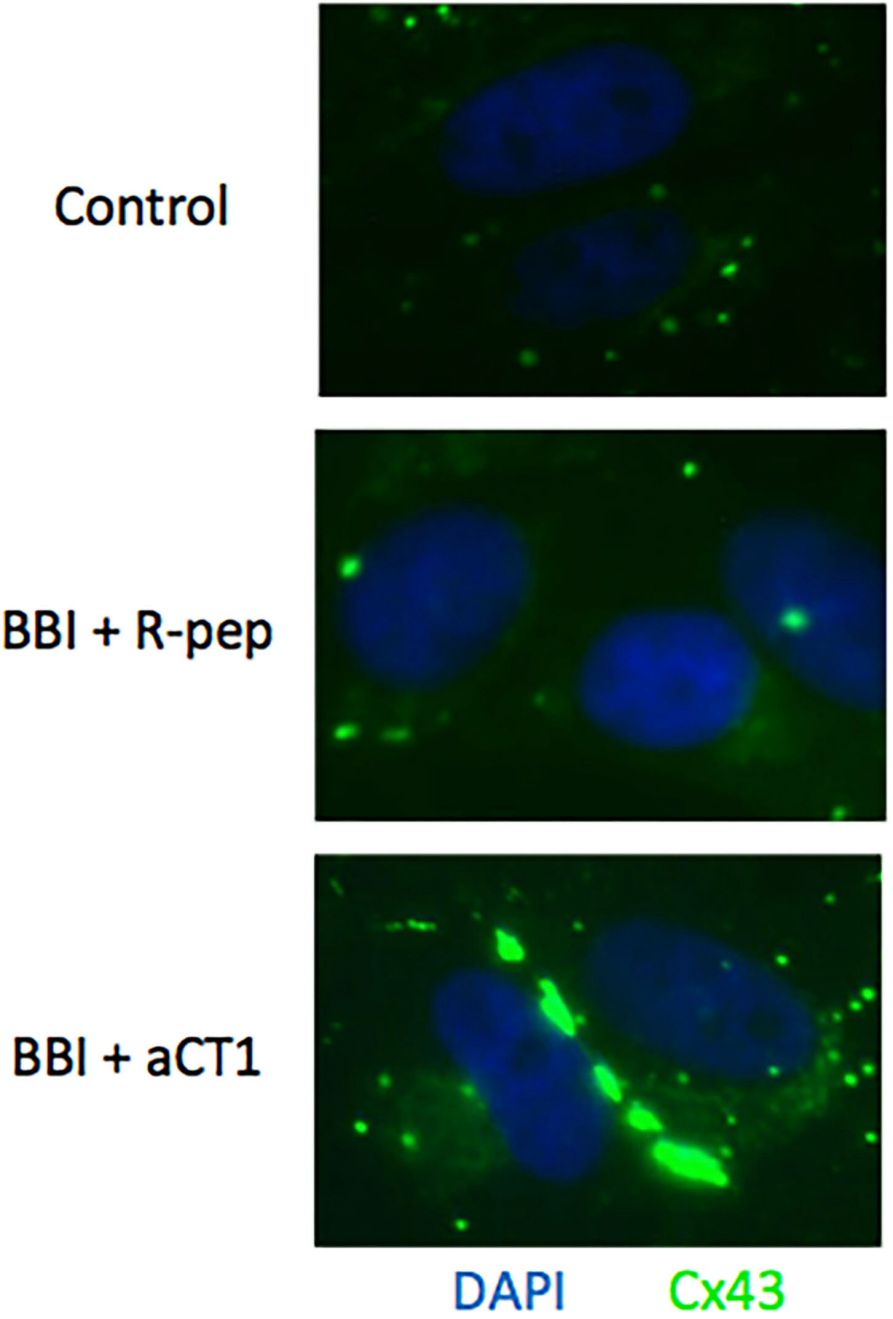
Cx43 expression after BBI and aCT1 peptide treatment in TLM1 cells. Cx43 plaques seen as green dots in the membrane of TLM1 cells submitted to treatment with 400 μg/ml BBI and 200 μM aCT1 peptide (or R-pep) for 3 days.

Immunofluorescence was carried out to compare the Cx43 localization in BBI and aCT1 treated cells with BBI and R-pep treated cells. As shown in Figure 5, combination treatment with BBI and aCT1 increased Cx43 expression compared with the combination treatment of BBI and R-pep in the cell membrane in the TLM1 cells, and a strong positivity to connexins was seen in the cell membranes.

## Discussion

Cell viability assays were carried out to evaluate the effects of aCT1 peptide in canine OMM TLM1 cells by treating them with 200 μM aCT1 peptide or R-pep. aCT1 peptide alone showed no effect on cell viability in the TLM1 cells. Additionally, we pre-tested the different treatment periods (2–4 days) of the peptide, and there were no significant differences on the cell viability (data not shown). An assay tested different concentrations of aCT1 peptide (0–400 μM), but the outcomes were similar to Figure 1 (data not shown). It is reported that aCT1 peptide (200 μM) inhibited human breast cancer cell proliferation in an *in vitro* study (33). The effect of aCT1 peptide may depend on the cell types. Murphy et al. (34) have also reported the possibility that different effects of aCT1 are shown in several cell lines. It was thought that in the absence of or with minimal Cx43 expression in the cell membrane, the aCT1 peptide could not exert its effect. In this context, Alaga et al. (40) revealed that Cx43 was detected in intracellular compartments, but not assembled in the gap junctions, and they suggested that the melanocytes do not form the Cx43 homocellular gap junctions. Although Cx43 levels increase during melanoma progression, connexin rarely assembles in gap junction structures (40). Therefore, we focused on a component, BBI, which may induce Cx43 expression.

BBI induced the expression of Cx43 genes in mice with M5076 ovarian tumor and decreased the tumor growth in this *in vivo* model (38). A similar effect of BBI was demonstrated in an osteosarcoma cell line (39). Tang et al. reported that treatment of prostate cancer cells (LNCaP) with 500 μg/ml BBI resulted in the inhibition of viability as measured in WST-1 assays, with the induction of Cx43 and expression of cleaved caspase-3 protein (41).

As shown in Figure 2, BBI treatment decreased cell viability in TLM1 cells at a pharmacological concentration (400 μg/ml). The mechanism has not been elucidated in detail in this study; however, it is known that BBI might improve the cell-to-cell communication because of its trypsin and chymotrypsin inhibitory activities. Furthermore, BBI has implications for Cx43 expression and induces apoptosis via factors such as a VEGF secretion inhibitory effect (42), besides the mitochondrial impairment and oxidative damage following proteasome 20S inhibition (43). These are reasons why BBI has been shown to have strong anti-carcinogenic activity in animal carcinogenesis model systems compared to other potential cancer chemopreventive agents in soybeans (44). Interestingly, a phase II clinical trial in patients with oral leukoplakia demonstrated a dose-dependent reduction in the oral lesion size after a 1-month treatment with BBI concentrates at doses of up to 1,066 CI units (45), and BBI is expected to prevent oral cancer (46). These findings have a potential for application in the development of new oral melanoma therapies.

Notably, as shown in Figure 3, the suppression effect of cell viability through the combination treatment of BBI and aCT1 peptide in TLM1 cells was remarkable. Moreover, immunofluorescence staining demonstrated that the combination treatment of BBI and aCT1 peptide induced a high expression of Cx43 in the cell membrane in TLM1 cells (Figure 4). These results indicate that Cx43 is important for canine OMM cell growth. Overall, the possibility of an enhancement effect of anticancer drugs by aCT1 peptide was emphasized similarly as in previous reports (33, 34); although further studies are needed to validate this finding. A finding that resveratrol enhances chemosensitivity in mouse melanoma model through Cx43 upregulation (47) supports this suggestion. Thus, Cx43 may influence the response of tumor cells to cancer therapies by facilitating the spread of antitumor drugs or death signals between neighboring tumor cells.

In conclusion, the findings of this study suggest that Cx43 upregulation may be useful for OMM treatment and warrants further research.

## Conclusions

In this study, it has been shown, for the first time, that the combined treatment with aCT1 peptide and BBI decreases cell viability in TLM1 canine melanoma cell line, which can possibly be used as a new therapy for canine oral melanomas.

## Data Availability Statement

The raw data supporting the conclusions of this article will be made available by the authors, without undue reservation.

## Author Contributions

AS executed the study and wrote the manuscript. IF and MN technical support and mentoring. GT technical support on cell cultures. RO technical support on immunocytochemistry. FH-B mentoring and technical support on immunocytochemistry. TY and EY mentoring and supply of materials. MD mentoring, financial support, manuscript writing, and editing. All authors contributed to the article and approved the submitted version.

## Conflict of Interest

The funder was not involved in the study design, collection, analysis, interpretation of data, the writing of this article or the decision to submit it for publication. The authors declare that the research was conducted in the absence of any commercial or financial relationships that could be construed as a potential conflict of interest.
